# The Agricultural Genome to Phenome Initiative (AG2PI): creating a shared vision across crop and livestock research communities

**DOI:** 10.1186/s13059-021-02570-1

**Published:** 2022-01-03

**Authors:** Christopher K. Tuggle, Jennifer Clarke, Jack C. M. Dekkers, David Ertl, Carolyn J. Lawrence-Dill, Eric Lyons, Brenda M. Murdoch, Nicole M. Scott, Patrick S. Schnable

**Affiliations:** 1 Iowa State University, Ames, USA; 2 University of Nebraska-Lincoln, Lincoln, USA; 3 Iowa Corn Growers Association, Johnston, USA; 4 University of Arizona, Tucson, USA; 5 University of Idaho, Moscow, USA

Predicting phenotype from genotype is a central challenge in biology. By understanding genomic information to predict and improve traits, scientists can address the challenges and opportunities of achieving sustainable genetic improvement of complex, economically important traits in agriculturally relevant species. Converting the enormous, recent technical advances in all areas of genomics and phenomics into sustained and ecologically responsible improvements in food and fuel production is complex. It will require engaging agricultural genome to phenome (G2P) experts, drawing from a broad community, including crop and livestock scientists and essential integrative disciplines (e.g., engineers, economists, data and social scientists). To achieve this vision, the USDA NIFA-funded project inaugurating the Agricultural Genome to Phenome Initiative (AG2PI) is working to: *Develop a cohesive vision for agricultural G2P research* by identifying research gaps and opportunities; *advancing community solutions* to these challenges and gaps; and *rapidly disseminating findings* to the broader community. Towards these ends, this AG2PI project is organizing virtual field days, conferences, training workshops, and awarding seed grants to conceive new insights (details at www.ag2pi.org). Since October 2020, more than 10,000 unique participants from every inhabited continent have engaged in these activities. To illustrate AG2PI’s scope, we present survey results on agricultural G2P research needs and opportunities, highlighting opinions and suggestions for the future. We invite stakeholders interested in this complex but critical effort to help create an optimal, sustainable food supply for society and challenge the community to add to our vision for future accomplishments by a fully actualized AG2PI enterprise.

## Differences hinder global advancements to sustainable food production

To meet the challenges and overcome the environmental stressors of agricultural production for an increasing global population, we must understand better how genotype begets phenotype and how the interactions of genotype and the environment influence agriculturally important, complex traits in crops and livestock. Advances in genetics, genomics, engineering, and agricultural, computational, and data sciences can potentially address these challenges. For example, while genome sequencing is now routine even for the large genomes in agriculturally important species, the functional components of these genomes are still largely unknown. Further, engineers have developed new technologies (e.g., robots and sensors) to measure novel biological and environmental variables at high resolution and/or at scale, and data scientists are developing approaches to analyze many types of very large (big data) datasets. Collaboration with agricultural scientists can focus such engineering and big data analytical approaches on analysis of phenotypes and environmental data that will facilitate an understanding of gene function and address fundamental problems of agricultural productivity and sustainability. However, the agricultural genomics and phenomics communities lack a cohesive vision on how to exploit these advances and opportunities, especially where cross-kingdom interactions and interdisciplinary synergies with other fields such as engineering and data science could lead to systems-level solutions [1]. To address this, the US Congress established AG2PI in the 2018 Farm Bill (https://www.congress.gov/bill/115th-congress/house-bill/2).

The crop and livestock research communities (including poultry and aquaculture) have separately begun to address some of these challenges [2–5]. While the needs of these communities have many commonalities and thus working together holds great potential, there are also significant differences between them. Biology drives some of these differences. For example, plants are sessile, while animals generally are not, and, therefore, plants are hardwired for resistance to some environmental stresses such as drought and heat. Although animals can and do respond to environmental changes, plants exhibit a greater degree of phenotypic plasticity in response to environmental changes, and, as a result, the crop genetics community has focused on accounting for GxE in its predictive models. Nevertheless, GxE is also of importance in livestock, especially when considering the generally high-health conditions in breeding herds compared to commercial production farms.

Differences between crop and livestock research communities are also driven by differences in the associated commercial sectors. For example, a handful of major crop breeding companies provide most of the hybrid corn seed to ~ 80,000 independent US farmers [6]. Traditionally, these companies have not shared information with each other, farmers, or academics. Similar restrictions to data availability apply to swine, poultry, and aquaculture industries, but less so for those concerned with sheep, beef, and dairy cattle. However, academic crop researchers can cost-effectively generate much of their own data. This provides flexibility with respect to experimental design, but financial constraints still often limit the scope of projects [4]. Currently, the ability to generate public genomic/phenomic research data is much more limited in livestock, with some exceptions. For beef and dairy cattle, large databases exist that contain phenotypic trait data from individual animals routinely recorded on production farms; and with certain restrictions, these data are available for research. Combined with the high value of individual animals that contribute significantly to the gene pool and justifies high-density genotyping of such individuals, this has enabled the creation of phenotypic and genomic databases on millions of individuals, offering substantial G2P research opportunities [7]. These collaborations and data sharing even extend across national borders [8].

The crop and livestock research communities are also organized at multiple levels. In both communities, there are many organizations focused on individual species that collectively represent the diversity of species. As well, there are also organizations that represent all crop and livestock commodities (e.g., National Animal Genome Research Program (NAGRP, NRSP-8), American Society of Plant Biology, Crop Science Society of America, and Functional Annotation of Animal Genomes (FAANG)) [9]. Both the crop and livestock research communities have well-established partnerships with industry/commodity groups and individual companies. We suggest that this network of interacting groups, often using similar methods in genetic improvement (see below), could form the basis for an alliance across crop and livestock communities to address G2P. An important AG2PI goal is to identify synergistic opportunities while recognizing the unique needs of each community.

## Identifying and strengthening similarities to address challenges

Several methodological similarities exist between the crop and livestock G2P communities, including common tools for molecular genetics and genomic analytics. Although computational strategies have diffused widely across both communities, encouragingly, over the past decades, there has been cross-fertilization between the crop and livestock breeding communities [10, 11], especially in statistical genetics. For example, methods for genetic evaluation such as best linear unbiased prediction (BLUP) and genomic prediction were initially developed by livestock breeders but then adopted by crop breeders [12]. Reciprocally, crop scientists have led the integration of biological models (e.g., crop growth models) into genomic prediction [13]. There are also specific opportunities for direct interaction between crops and livestock research that can be brought forward. For example, crop genotypes have the potential to affect livestock phenotypes through their use as feed (e.g., increased digestibility of silage enhances efficiency and sustainability of livestock production while potentially reducing greenhouse gas emissions). Similarly, the ability to integrate crop sciences with microbiome studies can lead to improved human health, suggesting similar potential with livestock [14].

While specific scientific goals of the agricultural G2P research communities exhibit some overlap in methods, their needs dovetail when accessing computational and data management resources and advanced data analytics. The two communities share requirements for integration of multiple sources and types of data; access to advanced cyberinfrastructure and data science expertise; development of novel sensors, robotics, and imaging platforms; and sharing of best practices and data. The two communities also utilize shared public resources such as NCBI for depositing genetic data and CyVerse for access to cyberinfrastructure for sharing private research data and running large-scale computational analyses. Although some computational resources exist that serve both plant and animal research [15], these communities often have specialized data resources for their organisms, which leads to some siloing of knowledge and information. Examples include Phytozome [16], which has a narrow, plant-specific focus; MaizeGDB [17] is concerned only with the crop plant and model organism *Zea mays* ssp. *mays* but includes all aspects of maize biology; and the Bovine Genome Database [18] covers only the genome annotation and variation of domestic cattle.

Additionally, these G2P research communities face similar challenges with regards to accessing emerging technologies such as remote sensing and edge computing capabilities, integrating large and heterogeneous datasets across the G2P spectrum, and rapidly reusing new algorithms for machine learning, bioinformatics, and predictive modeling of genotypes and/or phenotypes. When an advancement develops in a specific life science research area, there is often a lag before it is translated and adapted by other communities. Thus, there is a unique and timely opportunity to address these challenges by bringing together the crop and livestock G2P research communities to share best practices, identify common analytical pain-points, and co-develop solutions. A coordinated effort to increase communication among these communities will enable expansion of G2P research teams, including those from engineering, computational, and data sciences, who often seek new, high-impact challenges.

Importantly, the crop and livestock G2P communities also share the need to train and facilitate the flow of information to agricultural genomics researchers and stakeholders for global agricultural and societal benefits. Training this crucial workforce includes common methods and goals. For example, for the past ~ 20 years, the Iowa State University Animal Breeding and Genetics group has organized annual postgraduate short courses that are attended by both crop and livestock scientists. Also, a substantial number of Ph.D.s trained in animal breeding and genetics have been hired by the plant breeding industry in the past two decades. Other examples include the expansion of the plant-focused iPlant resource to CyVerse, which now services cyberinfrastructure needs across the life sciences generally, including both crop and livestock genomics (https://cyverse.org); and meetings such as the Gordon Research conferences on Quantitative Genetics and Genomics and the Plant and Animal Genome conference (PAG) serve both communities.

## AG2PI: goals, activities, and expected outcomes

While crop and livestock breeding programs have traditionally been organized very differently, the opportunities described above are bringing these fields closer together [19]. However, we cannot overlook the challenges and opportunities we have before us to use genomics and phenomics to improve agricultural outcomes for all stakeholders. We must recognize that the intricacy of these challenges and opportunities should not be minimized, and solving complex problems requires the successful integration of diversity of thought and expertise. While group intelligence for solving problems may not always transcend the limits of the single members, one thing is clear: high expertise in the absence of collaborative planning decreases team performance [20].

Two goals of AG2PI are to strengthen the ties between and among crop and livestock G2P research communities and to identify a shared vision for G2P research where common problems can be solved together. AG2PI’s overall goal is to assemble a transdisciplinary research community and to prepare this community for an anticipated, large-scale, agricultural G2P research and development effort to address sustainable genetic improvement of crops and livestock. To meet these goals, we are undertaking three highly interrelated objectives:

1. Develop a vision for agricultural G2P by identifying research needs and opportunities, as well as gaps in physical infrastructure and data management.
2. Foster first steps towards the development of community solutions to challenges identified in objective 1.
3. Communicate and disseminate findings of all activities through white papers, websites, and other scientific publications.

At the anticipated completion of the inaugural AG2PI project in late 2023, we expect to have facilitated several outcomes: (1) assembly and development of a research community prepared to tackle agricultural G2P (2); definition of needed infrastructure, resources, and protocols to conduct agricultural G2P research; and (3) concept papers that help define the future of agricultural G2P.

To address this multifaceted challenge, it is necessary to engage the expertise of crops and livestock researchers, those in broader scientific areas (e.g., engineering and data sciences), as well as industry. Organizations participating in AG2PI span institutions, nations, and industry, enabling their diverse members to become aware of and gain access to ideas, methods, and tools, while still in the proof-of-concept and early testing stages. AG2PI will also leverage existing networks to extend the reach of its activities. This will: (1) speed knowledge transfer, (2) create and reinforce an environment of inclusion by recognizing prior perspectives and achievements, and (3) identify challenges that these communities can address together.

**Fig. 1:**
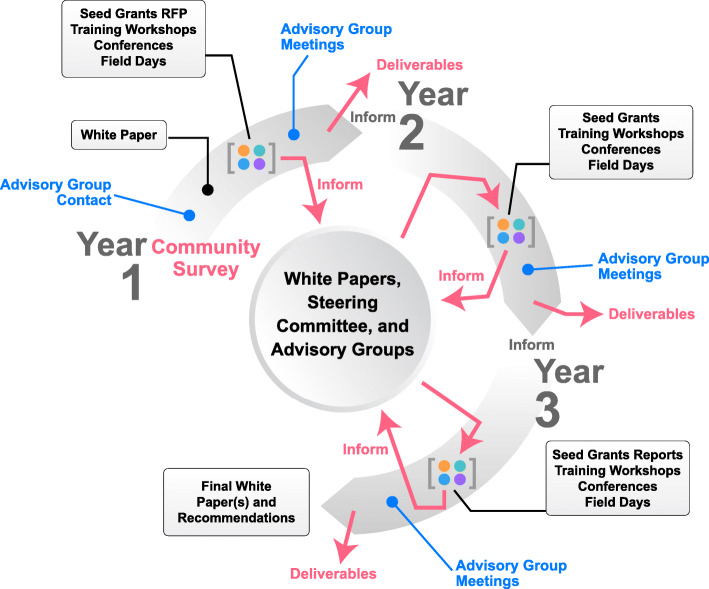
Proposed activities and projected timeline for AG2PI

To further develop this transdisciplinary community, we sponsor and coordinate virtual field days, conferences, training workshops, and award seed grants. Figure 1 illustrates a timeline of these activities. In most cases, activities are recorded and available for viewing on the AG2PI website for asynchronous participation.

## Field days

To expose the G2P community to diverse research activities, resources, and challenges across crops and livestock, the AG2PI project hosts virtual “field days.” These monthly events highlight research methods, approaches, and capacities and identify research gaps and challenges. Topics are presented at a level that allows a diverse audience to find a point of correspondence with their own research interests and challenges. Speakers have represented all career levels from undergraduate students to senior career scientists and industry stakeholders. See www.ag2pi.org for recordings and upcoming field day topics.

## Training workshops

To build technical strengths and future collaborative G2P communities, the AG2PI project facilitates workshops for researchers and stakeholders from all backgrounds and computational skill levels to develop best practices, common vocabularies, and technical expertise around genomic and phenomic cyberinfrastructure, data tools and pipelines, statistics, and experimental techniques. For workshop delivery, AG2PI works with its partner organizations to leverage their existing expertise and training materials. One workshop or series is organized every month, building a suite of interconnected training opportunities and allowing the agriculture G2P community to develop baseline technical skills, gain expertise in teaching others, and further competencies for advanced computational analyses and FAIR data principles [21]. The workshops are virtual to increase accessibility and participation. Both short- and long-format workshops are provided with an eye towards improving agriculture G2P participation for nontraditional students. Short-format workshops begin with a lecture followed by a hands-on/interactive session and a discussion of strengths, limitations, best practices, and areas for future development. Long-format workshops help onboard and increase the skill level of people who are new to computationally intensive science.

## Seed grants

The AG2PI Seed Grant program is designed to promote collaboration and the development and cross-pollination of tools, data, and ideas to enable and facilitate future agriculture G2P research across disciplines, species, and sectors. Seven grants were awarded in the first round of funding (March 2021), ranging from $15,000 to 20,000, with project durations of 6–12 months. Funded project summaries are available at www.ag2pi.org. The project leaders of these seed grants represented 15 institutions including USDA-ARS, land grant universities, and Historically Black Colleges and Universities. Project deliverables include publicly available datasets, training materials, workshops, machine learning simulations, publications, conference presentations, and an online catalog of the available resources for data science in animal and plant agricultural genomics. Additional rounds of funding are planned, with requests for proposals at three levels of maturity:

1. *Emerging grants* (6–12 months in duration, up to $20,000). These awards will be similar in scope to the awards from the initial round of AG2PI seed grants.
2. *Enabling grants (*12 months in duration, up to $50,000). These awards will be aimed at enlarging the scope or supporting further development of a project that has successfully completed an emerging grant or a non-AG2PI project at a similar stage of development.
3. *Established grants* (12–18 months in duration, up to $75,000). These awards will be aimed at providing support towards long-term sustainability and ability to enable cross-kingdom agricutural G2P research and engagement of a project that has successfully completed an enabling-like project or of a non-AG2PI project at a similar stage of development.

## Conferences and surveys

The goals of AG2PI conferences are to identify gaps in knowledge, infrastructure, protocols, and coordination, as well as to highlight opportunities for cross-kingdom collaborative research and education within the crop and livestock G2P communities. The first conference focused on promoting the AG2PI Seed Grant program. This virtual conference included a teaming event to introduce investigators from the crop, livestock, data, and social sciences to each other so they could potentially collaborate on an AG2PI seed grant proposal. Subsequently, the AG2PI project has offered conferences as listening sessions in conjunction with established national or international conferences. Surveys within these listening sessions further identify agricultural G2P challenges that could be worked on together to overcome. Participants were asked to identify existing gaps in agriculture G2P research, community-building efforts and needs, and to brainstorm opportunities to address the challenges that have led to these gaps.

**Fig. 2:**
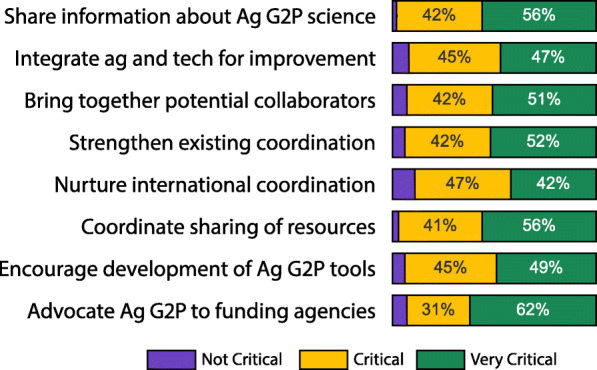
Survey responses to the question “How important do you believe each of the following activities are for advancing agricultural G2P research?”

An online survey was conducted in 2020 to obtain comprehensive input from the agricultural community on the challenges to accelerate agricultural G2P research and specific activities that would address those challenges. A total of 467 people provided input to a variety of questions. They represented academics, government, and industry, and covered crop and livestock genetics/genomics, data and computer sciences, engineering, physics, economics, and social science*.* While the majority of respondents were from the USA (69%), all six inhabited continents were represented. One question asked which community activities are important for advancing agricultural G2P research. While there was strong support for all AG2PI activities shown in Fig. 2, the respondents identified coordinating the sharing of resources and information as most critical.

To tailor AG2PI activities to the needs and interests of its stakeholders, information on how the AG2PI community wished to participate was also gathered. When survey participants were asked what training workshops were so crucial that they were willing to help organize or teach, learning modern genetics/genomics as well as bioinformatics were rated highest. Other specific AG2PI activities also had strong support, with those of highest interest being: (1) holding single-subject, in-depth open house “field days” on agricultural G2P methods and approaches, (2) organizing virtual community-wide agricultural G2P conferences, and (3) surveying community opinion on agricultural G2P needs, as we have initiated. Surveys will continue in order to improve our efforts and communicate identified needs to stakeholders and funding agencies.

## Cooperation and institutional involvement

The inaugural AG2PI project has engaged a diverse group of “institutional partners” that collectively represent much of the breadth of agricultural G2P research. The list of participating partner organizations is at: https://www.ag2pi.org/institutional-involvement/. Institutional partners help open communication channels between AG2PI and the larger community and contribute to project activities by providing materials or expertise. For example, CyVerse hosts many of the project’s training workshops. AG2PI’s partnership with CyVerse is helping to advance common goals including lowering the barriers to FAIR data management and analytics. Other AG2PI partner organizations, such as the FAANG Consortium (Functional Annotation of Animal Genomes, https://www.animalgenome.org/community/FAANG/) and USDA ARS (https://www.ars.usda.gov/), have led virtual field days. Others, such as the National Animal Genome Research Program (NAGRP, https://www.animalgenome.org/) and the North American Plant Phenotyping Network (NAPPN, http://www.plantphenotyping.org) provide communication across the livestock genetics and plant phenotyping research communities, respectively.

The AG2PI project is advised by a Scientific Advisory Board comprised of knowledgeable thought leaders in areas relevant to our mission (see membership at https://www.ag2pi.org/people/scientific-advisory-board/). These areas include genetics, crop and livestock breeding, genomics, phenomics, statistics, machine learning, data science and security, rural sociology, agricultural economics, and training.

## Future outcomes from AG2PI

The AG2PI project expects to demonstrate significant need for increased research and development of tools enabling crops and livestock to sustainably provide more and better foods and fuels for domestic and global consumers. This will be achieved by identifying and documenting scientific, economic, and societal advances, and realization of the potential for practical applications of agriculture G2P discoveries. This will enable the USA to increase the overall efficiency of agricultural products, thereby improving both profitability and sustainability of USA agriculture, with parallel impacts at a global scale.

Our expected outcomes through the four activities described above include:

- *Advancing data and tools*: Comprehensive, public datasets of genomes and traits across many species of crops and livestock, as well as best practices to store and display these data to the public, will be made available. We expect AG2PI to demonstrate the need for and identify methods to predict future crop and livestock traits, starting with genomic information, and to create publicly available, user-accessible tools for analyzing new datasets.
- *Training for innovation*: New technologies and data-driven decision making for agricultural systems will require and create opportunities for new types of training and educational programs, from which a more technically literate agricultural workforce can emerge. We expect AG2PI to enhance the emergence of a new generation of scientists trained to use agriculturally relevant datasets and tools, and educators who can share these new technologies with industry stakeholders for uptake to ensure the continued profitability of food chains.
- *Confronting climate crises*: In a changing climate, it is important to consider how modifying agricultural production methods can contribute toward reducing the carbon footprint while continuing to meet the food, fuel, and fiber societal needs. Development and genetic improvement of crops and livestock that are not only more efficient but can thrive in changing environments is vital. Cross-fertilization of ideas across the agriculture spectrum through AG2PI is expected to advance future-proofing of agricultural systems against impacts of climate change.
- *Promoting equity*: The ultimate goals of AG2PI are to develop technologies that enable development of more climate-resilient, sustainable, and desirable crops and livestock, and to generate evidence-based management tools that can be readily applied to a wide variety of crops and livestock species in diverse production systems. Public development and discussion of such technologies will help level the playing field among farms across scales and democratize information and knowledge to benefit society.

To achieve the goals outlined above, the current AG2PI project will continue to engage the following external stakeholders, advocates, and other relevant groups to garner distinct benefits from these and future AG2PI efforts:

1. Agricultural scientists and engineers will benefit from identifying and solving challenges that impede basic research and understanding how to translate their discoveries to relevant industry applications.
2. Livestock and crop breeding companies will benefit from technologies to increase the rate of genetic improvement of their animals and crops for traits relevant to a changing agricultural system and at lower costs per unit of gain.
3. Producers/farmers will benefit from increased profitability derived from improved crops and livestock and a more sustainable agriculture.
4. Consumers will benefit from a more stable and cost-effective supply of healthy food.
5. Society at-large will benefit from a more sustainable agriculture with associated environmental benefits.

These diverse stakeholders are engaged with AG2PI in various ways. Category 1 stakeholders are the targeted AG2PI participants. Categories 2–3 are represented on the AG2PI External Stakeholder Committee (https://www.ag2pi.org/institutional-involvement/). Categories 1 and 2 participated in the AG2PI survey described above. As AG2PI matures, its engagement with categories 4 and 5 will also increase.

## Recommendations for future action required to achieve this vision for AG2PI

To expand the current AG2PI activities described above, we propose that acceleration of the following efforts is urgently needed:

- *Coordination of AG2PI activities* with US federal government agencies in USDA (ARS, NIFA, HEP) and NSF, NIH, DOE, as well as internationally (EU, China, Japan, Australia, New Zealand, Brazil, India and others). One approach is an international consortium to nurture an organized effort to share methods and tools for data sharing, storage, as well as efficient and effective personnel training in data science.
- *Expansion of public–private partnerships* to increase communication with several facets of the agricultural genetics/genomics industry. The main purpose of such groups would be to identify common interests and methods for achievable application of current and future G2P knowledge.
- *Increased efforts to train students and workers* interested in agriculture, data science, and bioinformatics. A major bottleneck of applying agriculture G2P knowledge in many relevant fields is the lack of appropriately trained workers, requiring a dedicated effort to recruit and train individuals in agriculture.
